# Pulmonary artery to aorta ratio for the detection of pulmonary hypertension: cardiovascular magnetic resonance and invasive hemodynamics in heart failure with preserved ejection fraction

**DOI:** 10.1186/s12968-015-0184-3

**Published:** 2015-08-30

**Authors:** Gültekin Karakus, Andreas A. Kammerlander, Stefan Aschauer, Beatrice A. Marzluf, Caroline Zotter-Tufaro, Alina Bachmann, Aleks Degirmencioglu, Franz Duca, Jamil Babayev, Stefan Pfaffenberger, Diana Bonderman, Julia Mascherbauer

**Affiliations:** Department of Cardiology, Medical University of Vienna, Waehringer Guertel 18-20, 1090 Vienna, Austria; Acibadem Maslak Hospital, Istanbul, Turkey; Otto Wagner Hospital, Vienna, Austria

**Keywords:** Cardiovascular magnetic resonance, Pulmonary hypertension, PA:Ao ratio

## Abstract

**Background:**

Previous work indicates that dilatation of the pulmonary artery (PA) itself or in relation to the ascending aorta (PA:Ao ratio) predicts pulmonary hypertension (PH). Whether these results also apply for heart failure with preserved ejection fraction (HFpEF) is unknown.

In the present study we evaluated the diagnostic and prognostic power of PA diameter and PA:Ao ratio on top of right ventricular (RV) size, function, and septomarginal trabeculation (SMT) thickness by cardiovascular magnetic resonance (CMR) in HFpEF.

**Methods and Results:**

159 consecutive HFpEF patients were prospectively enrolled. Of these, 111 underwent CMR and invasive hemodynamic evaluation.

By invasive assessment 64 % of patients suffered from moderate/severe PH (mean pulmonary artery pressure (mPAP) ≥30 mmHg). Significant differences between groups with and without moderate/severe PH were observed with respect to PA diameter (30.9 ± 5.1 mm versus 26 ± 5.1 mm, *p* < 0.001), PA:Ao ratio (0.93 ± 0.16 versus 0.78 ± 0.14, *p* < 0.001), and SMT diameter (4.6 ± 1.5 mm versus 3.8 ± 1.2 mm; *p* = 0.008). The strongest correlation with mPAP was found for PA:Ao ratio (*r* = 0.421, *p* < 0.001). By ROC analysis the best cut-off for the detection of moderate/severe PH was found for a PA:Ao ratio of 0.83.

Patients were followed for 22.0 ± 14.9 months. By Kaplan Meier analysis event-free survival was significantly worse in patients with a PA:Ao ratio ≥0.83 (log rank, *p* = 0.004). By multivariable Cox-regression analysis PA:Ao ratio was independently associated with event-free survival (*p* = 0.003).

**Conclusion:**

PA:Ao ratio is an easily measureable noninvasive indicator for the presence and severity of PH in HFpEF, and it is related with outcome.

## Background

About 50 % of patients presenting with symptoms of heart failure are diagnosed with heart failure with preserved ejection fraction (HFpEF). HFpEF is characterized by impaired diastolic function due to abnormal relaxation of the left ventricle (LV) as well as increased chamber stiffness [1]. The pathophysiology underlying HFpEF is still incompletely understood. Arterial hypertension, coronary artery disease and diabetes mellitus seem to play an important role [2]. The development of post-capillary pulmonary hypertension (PH) is a common feature of HFpEF and associated with substantial morbidity and mortality, frequent hospitalizations and an impaired quality of life [3–6].

Accurate assessment of PH requires invasive measurement of pulmonary artery (PA) pressure and pulmonary arterial wedge pressure (PAWP) [7]. Because of the invasive nature of this procedure the majority of HFpEF patients today are not evaluated by right heart catheter (RHC). Thus, diagnosis of HFpEF-PH mostly relies upon echocardiography. The estimation of PA pressure by echocardiography is a routine measurement, however, the accuracy of this method is limited [8] and the echocardiographic estimation of pulmonary vascular resistance is unreliable [9]. Therefore, an alternative straightforward non-invasive technique in addition to echocardiography is desirable to predict the likelihood of PH in HFpEF patients.

Cardiovascular magnetic resonance (CMR) is the current gold-standard technique to assess right heart dimensions, function, wall thickness, as well as the dimensions of the great arteries [10]. Previous work shows that dilatation of the pulmonary artery (PA) itself or in relation to the diameter of the ascending aorta (PA:Ao ratio) predicts PH [11–15]. Furthermore, right ventricular septomarginal trabeculation (SMT) mass has been identified as a CMR-derived marker of PH [16].

The present study was intended to determine the usefulness and prognostic significance of easily measureable variables such as PA diameter and PA:Ao ratio on top of CMR derived right ventricular (RV) dimension, ejection fraction, SMT dimensions, and RV free wall thickness (RVFW) in a prospective, well defined HFpEF cohort.

## Methods

### Study design

This was a prospective observational study performed at the Medical University of Vienna. All participants gave written informed consent. The institutional review board approved the study protocol (EK #796/2010).

### Patients

Consecutive patients with suspected HFpEF-PH were invited to participate. HFpEF was diagnosed in the presence of: (1) symptoms or signs of heart failure; (2) normal or mildly reduced left ventricular (LV) systolic function (LV ejection fraction (EF) >50 %); (3) evidence of abnormal LV relaxation or diastolic stiffness (E/e’ > 8 by echocardiography; and (4) NT-proBNP serum levels ≥220 pg/mL [17]. Reasons for exclusion included pacemaker or other conditions precluding patients from CMR, regional wall motion abnormalities, significant coronary artery disease, significant valvular or congenital heart disease, or a glomerular filtration rate (GFR) below 30 mL/min/1.73m^2^. All patients underwent ventilation-perfusion scans and lung function testing including diffusing capacity of the lung for carbon monoxide to rule out chronic thromboembolic PH and PH due to significant lung disease.

### Right and left heart catheterization

PAWP, mean pulmonary artery pressure (mPAP), and cardiac output (CO) were determined. CO was measured by both thermodilution and Fick method. Simultaneously, all patients underwent direct assessment of LV filling pressures, followed by coronary angiography. Derived hemodynamic parameters were calculated according to standard formulae [18]. PH was diagnosed according to recent guidelines as mPAP ≥ 25 mmHg at rest [19]. PH was classified as moderate or severe when the mPAP was ≥30 mmHg. The transpulmonary pressure gradient (TPG) was defined as difference between mPAP and PAWP and the diastolic pressure gradient (DPG) as difference between diastolic PA pressure and PAWP.

### Cardiovascular magnetic resonance

All CMR studies were performed on a 1.5-T scanner (Avanto, Siemens Medical Solutions, Erlangen, Germany) within 30 days of RHC (mean 14.8 ± 9.0 days). Studies consisted of functional and Late Gadolinium Enhancement (LGE) imaging, according to standard protocols [20]. For cine imaging, steady state free precision (SSFP) images were used (repetition time 3.2 ms, echo time 1.2 ms, flip angle 64°, voxel size 1.4 × 1.4 × 6 mm, matrix 180×256). For LGE imaging of the left ventricle, segmented inversion recovery sequences (repetition time: 700 ms, echo time 1.22 ms, flip angle 50°, voxel size 1.4 × 1.4 ×. 8 mm, matrix 146×256) were acquired 10 min after injection of 0.1 mmol/kg gadolinium-DTPA (Gadovist®; Bayer Vital GmbH, Leverkusen, Germany).

**Fig. 1:**
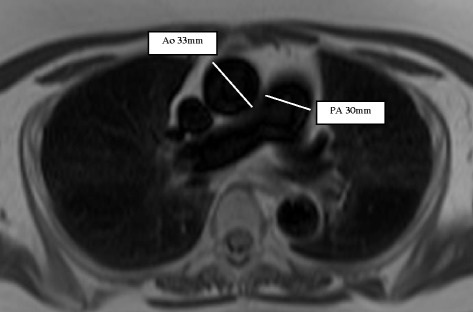
Example for the measurement of pulmonary artery (PA) and ascending aorta (Ao) diameters in the axial black blood sequence in a patient with a mean pulmonary artery pressure of 33 mmHg

PA and Ao diameters were measured from inner-edge to inner-edge in axial half-Fourier single-shot turbo spin-echo (HASTE) black blood images (repetition time 810 ms, echo time 43 ms, flip angle 160°, voxel size 1.6 × 1.6 × 5 mm, matrix 192×256) at the level of the PA bifurcation (Fig. 1).

**Fig. 2:**
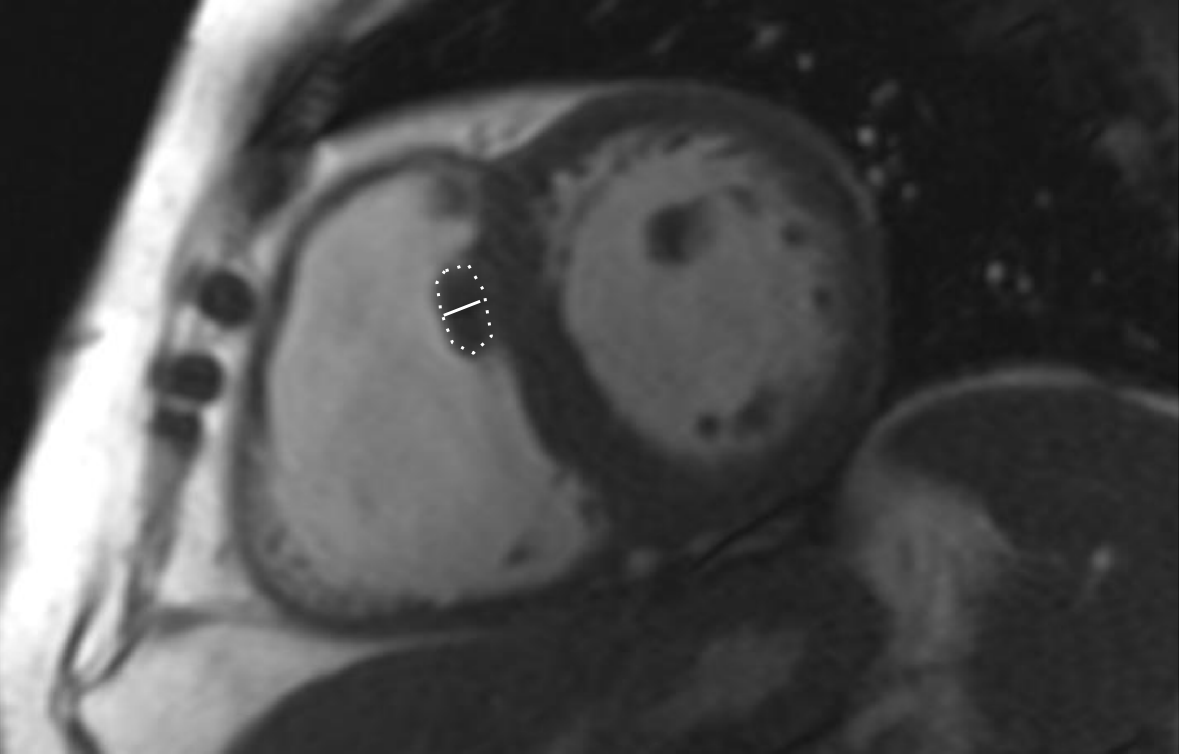
Example for the measurement of the septomarginal trabeculation (SMT) diameter and area in an axial cine image in a patient with a mean pulmonary artery pressure of 33 mmHg

For SMT measurements, cine images were analyzed. The largest area and the longest medio-lateral diameter of the septal insertion part of SMT as identified in the second or third basal slice of the short axis cine stack were measured (Fig. 2). The RVFW was measured in end-diastole in the same short axis image used for SMT quantification.

All CMR studies were read by two independent observers blinded to clinical data (GK, SP and JB).

### Outcome measures

Patients were prospectively followed by ambulatory visits and telephone calls at 6-month intervals. The main outcome measure was a combined endpoint consisting of hospitalization for heart failure or death from cardiac causes. Endpoints were ascertained by follow-up visits and phone calls.

### Statistical analysis

Categorical data are presented as total numbers or percent, and continuous variables as mean ± standard deviation. Chi square test or Fisher´s exact test were applied for categorical variables and the Wilcoxon two-sample test for continuous variables. Kaplan-Meier estimates were used to calculate cardiac event rates. Differences between Kaplan-Meier curves were analyzed using the log rank test.

Binary logistic regression analysis was used to identify variables associated with moderate/severe PH (mPAP ≥30 mmHg). In addition, a univariable Cox regression analysis was used to identify parameters (SMT diameter, SMT area, RVFW, PA size and PA:Ao ratio) associated with event-free survival. In a second step these parameters were adjusted for potential cofounding clinical variables such as age, sex, atrial fibrillation, arterial hypertension, diabetes, chronic obstructive pulmonary disease, and coronary artery disease. All variables with a *p*-value <0.05 in the univariable model were entered into a multivariable model using a stepwise approach. Hazard and odds ratios for the PA:Ao ratio were reported as PA diameter divided by Ao diameter times 100. Receiver Operating Characteristic (ROC) curves were used to determine cut-off values for the detection of moderate/severe PH. Interobserver variability was described by using Intra Class Correlation Coefficients (ICCs). Statistical analyses were performed with SPSS Statistics version 18 (IBM, Armonk, New York). Results were considered statistically significant if *p*-values were <0.05.

## Results

### Baseline characteristics

Between 2010 and 2013 159 consecutive patients were enrolled. Of these, 48 had to be excluded from the study because CMR was not possible due to pacemakers, GFR <30 mL/min/ 1.73 m^2^, or claustrophobia. Of the remaining 111 patients (32.4 % male, 70 ± 9 years old) who underwent CMR, 64 % suffered from moderate or severe PH (mPAP ≥ 30 mmHg by RHC). Clinical characteristics, hemodynamic measurements, and CMR parameters of registered patients are summarized in Table 1.

**Table 1:** Baseline patient characteristics

Variable	mPAP < 30 mmHg 40 patients (36 %)	mPAP ≥ 30 mmHg 71 patients (64 %)	All 111 patients	*p* value
Clinical parameters				
Age [years]	68.9 ± 10.6	70.6 ± 8.4	70.0 ± 9.3	0.348
Male sex [%]	32.5	32.4	32.4	0.991
BSA [m^2^]	1.9 ± 0.3	1.9 ± 0.2	1.9 ± 0.2	0.367
AFib [%]	51.3	67.1	61.5	0.103
Diabetes [%]	23.1	50.0	40.4	**0.006**
HbA1c [%]	5.9 ± 0.6	6.3 ± 1.0	6.2 ± 0.9	**0.028**
Smoking [%]	33.3	43.3	39.6	0.312
CAD* [%]	5.1	27.1	19.3	**0.005**
Hypertension [%]	94.9	100.0	98.2	0.056
BMI [kg/m^2^]	29.3 ± 6.3	31.4 ± 6.0	30.7 ± 6.1	0.079
Heart rate [beats/min]	72.2 ± 14.8	70.8 ± 13.3	71.4 ± 13.8	0.626
6-MWD [m]	396.7 ± 104.3	301.0 ± 110.2	334.4 ± 117.0	**<0.001**
GFR [mL/min/1.73m^2^]	64.9 ± 22.7	59.10 ± 17.4	61.0 ± 19.5	0.142
NT-proBNP [pg/ml]	1363.8 ± 2127.4	2252.1 ± 3746.1	1934.8 ± 3278.6	0.171
NYHA				0.081
II [%]	41.0	23.2	29.6	
III [%]	56.4	66.7	63.0	
IV [%]	2.6	10.1	7.4	
Hemodynamic parameters				
mPAP [mmHg]	23.9 ± 3.6	39.1 ± 8.0	33.7 ± 10.0	**<0.001**
sPAP [mmHg]	36.7 ± 6.1	61.5 ± 15.4	52.6 ± 17.5	**<0.001**
dPAP [mmHg]	15.9 ± 4.0	25.3 ± 6.2	21.9 ± 7.1	**<0.001**
PAWP [mmHg]	15.5 ± 3.3	22.1 ± 4.3	19.7 ± 5.1	**<0.001**
LVEDP [mmHg]	16.1 ± 4.6	22.4 ± 6.0	20.3 ± 6.3	**<0.001**
PVR [dyne*sec/cm^5^]	134.8 ± 67.4	276.9 ± 125.1	226.5 ± 127.7	**<0.001**
PAC [ml/mmHg]	3.9 ± 2.1	2.3 ± 0.9	2.9 ± 1.7	**<0.001**
CO [l/min]	5.3 ± 1.5	5.2 ± 1.2	5.2 ± 1.3	0.829
Cardiovascular magnetic resonance imaging parameters				
LV EDD [mm]	46.5 ± 4.7	47.8 ± 6.3	47.3 ± 5.8	0.259
LV EDV [ml]	117.7 ± 33.5	133.5 ± 52.6	127.9 ± 47.1	0.057
LV EDVi [ml/m^2^]	62.4 ± 18.4	68.3 ± 23.7	66.2 ± 22.0	0.160
LV EF [%]	62.7 ± 10.6	63.3 ± 11.9	63.1 ± 11.4	0.813
LV mass [g]	108.4 ± 37.6	117.5 ± 36.1	113.0 ± 36.8	0.250
IVS [mm]	11.5 ± 2.6	11.6 ± 2.2	11.5 ± 2.3	0.924
LA diameter [mm]	62.9 ± 8.7	66.7 ± 9.3	65.3 ± 9.2	**0.035**
LA area [cm^2^]	28.3 ± 6.8	33.5 ± 10.2	31.6 ± 9.4	**0.005**
RV EDD [mm]	38.9 ± 5.9	40.8 ± 8.1	40.1 ± 7.4	0.193
RV EDV [ml]	143.4 ± 39.6	160.0 ± 67.1	151.3 ± 51.7	0.109
RV EDVi [ml/m^2^]	76.1 ± 20.7	80.5 ± 29.2	78.9 ± 26.4	0.453
RV EF [%]	50.9 ± 9.1	52.5 ± 11.3	51.9 ± 10.6	0.452
RA diameter [mm]	63.5 ± 8.7	66.7 ± 8.9	65.5 ± 9.0	0.072
RA area [cm^2^]	28.3 ± 8.9	30.5 ± 8.8	29.7 ± 8.9	0.221
LGE present [%]	25.0	34.8	31.2	0.288
Amount of LGE*** [%]	6.8 ± 2.7	9.6 ± 4.7	8.8 ± 4.3	0.093
Subendocardial [%]	0	5.8	3.7	0.294**
Midmyocardial [%]	2.5	10.1	7.3	0.254**
RV insertion point [%]	22.5	18.8	20.2	0.646

*BSA* indicates body surface area, *AFib* atrial fibrillation, *CAD* coronary artery disease, *BMI* body mass index, *6-MWD* six minute walk distance, *GFR* glomerular filtration rate, *NYHA* New York Heart Association functional class, *mPAP* mean pulmonary artery pressure, *sPAP* systolic pulmonary artery pressure, *dPAP* diastolic pulmonary artery pressure, *PAWP* pulmonary artery wedge pressure, *LVEDP* left ventricular end-diastolic pressure, *PVR* pulmonary vascular resistance, *PAC* Pulmonary artery compliance, *CO* cardiac output, *LV* left ventricle, *EDD* end-diastolic diameter, *EDV* end-diastolic volume, *i* indexed to body surface area, *EF* ejection fraction, *IVS* interventricular septum thickness, *LA* left atrium, *RV* right ventricle, *RA* right atrium, *LGE* Late Gadolinium Enhancement

*non-significant coronary artery disease or prior revascularization

^**^ *p*-values derive from Fisher’s exact test

^***^Amount of Late Gadolinium Enhancement is given in % of total left ventricular volume

With respect to baseline clinical parameters, patients with moderate/severe PH more frequently presented with diabetes (50.0 % versus 23.1 %, *p* = 0.006), non-significant coronary artery disease/previous revascularization (27.1 % versus 5.1 %, *p* = 0.005); shorter 6-min walk distances (301 ± 110 m versus 397 ± 104 m, *p* < 0.001) and had more dilated left atria (LA; 66.7 ± 9.3 mm versus 62.9 ± 8.7 mm, *p* = 0.035 for diameters and 33.5 ± 10.2 cm^2^ versus 28.3 ± 6.8 cm^2^, *p* = 0.005 for areas).

Invasive assessment showed significant differences between patients with and without moderate/severe PH with respect to all measured variables except for CO, which was similar in both groups (5.2 ± 1.2 versus 5.2 ± 1.5 l/min, *p* = 0.829).

60 (54 %) patients had a TPG >12 mmHg (formerly classified as “out-of-proportion PH”) and 13 (12 %) had a DPG ≥7 mmHg, indicating combined pre- and postcapillary pulmonary hypertension [21].

Patients with a TPG >12 mmHg had a significantly higher PA:Ao ratio as compared to patients with a TPG ≤12 mmHg (0.9 ± 0.2 versus 0.8 ± 0.2, *p* = 0.001) whereas PA:Ao ratios did not differ between patients with a DPG of ≥ and <7 mmHg (0.9 ± 0.2 versus 0.9 ± 0.2, *p* = 0.230). However, this lack in statistical significance may be due to the small proportion of patients with DPGs ≥7 mmHg (12 % of the study cohort).

LGE was present in 31 % (*n* = 34) of our patients and involved 8.8 ± 4.3 % of left ventricular myocardial mass. In the majority (*n* = 22), LGE was present at the insertion point of the right ventricle; 8 patients had midmyocardial and 4 subendocardial enhancement. There were no significant differences in LGE pattern or degree between groups with and without moderate/severe PH.

### Dimensions of the great arteries and indices of right ventricular hypertrophy for the prediction of moderate/severe pulmonary hypertension

**Fig. 3:**
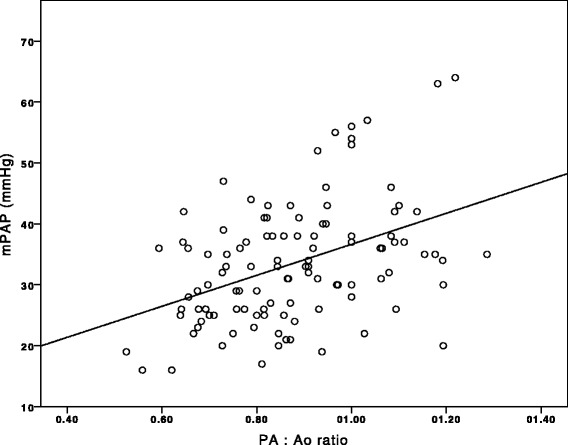
Scatter plot showing the correlation between pulmonary artery to ascending aorta (PA:Ao) ratio and mean pulmonary artery pressure (mPAP; *r* = 0.421, *p* < 0.001)

Dimensions of the great arteries and indices of RV hypertrophy by CMR are displayed in Table 2. Patients with moderate/severe PH showed significantly larger PA diameters than the comparator (30.9 ± 5.1 mm versus 26 ± 5.1 mm, *p* < 0.001) and also had significantly higher PA:Ao ratios (0.93 ± 0.2 versus 0.79 ± 0.1, *p* < 0.001). SMT diameters (4.6 ± 1.2 mm versus 3.8 ± 1.2 mm, *p* = 0.008) were larger in patients with moderate/severe PH, while areas did not differ between groups (*p* = 0.136). RVFW showed a wide range from 2.0 to 7.2 mm (4.2 ± 1.1 mm) and did also not differ between patients with and without moderate/severe PH (*p* = 0.183). PA:Ao ratio, PA diameter and SMT diameter were significantly correlated with mPAP (*r* = 0.421, *p* < 0.001; *r* = 0.401, *p* < 0.001; *r* = 0.267, *p* = 0.005; respectively). Fig. 3 displays the correlation of PA:Ao ratio with mPAP.

**Table 2:** Dimensions of the great arteries and indices of right ventricular hypertrophy for the prediction of moderate/severe pulmonary hypertension

Variable	mPAP < 30 mmHg 40 patients (36 %)	mPAP ≥ 30 mmHg 71 patients (64 %)	All 111 patients	*p*-value
PA diameter [mm]	26.3 ± 5.1	30.9 ± 5.1	29.2 ± 5.5	**<0.001**
Ao diameter [mm]	33.6 ± 4.3	33.4 ± 4.1	33.5 ± 4.1	0.856
PA:Ao ratio	0.79 ± 0.1	0.93 ± 0.2	0.89 ± 0.2	**<0.001**
SMT diameter [mm]	3.8 ± 1.2	4.6 ± 1.2	4.3 ± 1.4	**0.008**
SMT area [mm^2^]	0.96 ± 0.3	1.11 ± 0.5	1.06 ± 0.4	0.136
RVFW [mm]	4.0 ± 1.2	4.3 ± 1.1	4.2 ± 1.2	0.183

*PA* indicates pulmonary artery, *Ao* ascending aorta, *RVFW* right ventricular free wall, *SMT* septomarginal trabeculation

Table 3 shows the binary logistic regression model for predicting the presence of moderate/severe PH. By univariable analysis, SMT diameter (*p* = 0.010), PA diameter (*p* < 0.001), and PA:Ao ratio (*p* < 0.001) were associated with moderate/severe PH. However, by multivariable analysis only PA:Ao ratio remained independently associated with the presence of moderate/severe PH.

**Table 3:** Binary logistic regression model for the presence of moderate/severe pulmonary hypertension (mean pulmonary artery pressure ≥30 mmHg)

	Regr.Coeff.	*p*-value	OR	95 % CI for OR	
Univariable					
SMT area	0.853	0.090	2.346	0.876	6.282
SMT diameter	0.404	**0.010**	1.498	1.103	2.034
RVFW	0.236	0.200	1.267	0.882	1.818
PA diameter	0.175	**<0.001**	1.191	1.091	1.301
Ao diamter	−0.008	0.875	.992	0.903	1.090
PA:Ao x 100	0.066	**<0.001**	1.068	1.035	1.103
RVEDV	0.005	0.239	1.005	0.997	1.013
RVEF	0.017	0.377	1.018	0.979	1.058
Multivariable					
PA:Ao x 100	0.066	**<0.001**	1.068	1.035	1.103

*SMT* indicates septomarginal trabeculation, *RVFW* right ventricular free wall, *PA* pulmonary artery, *Ao* ascending aorta, *RVEDV* right ventricular end-diastolic volume, *RVEF* right ventricular ejection fraction

**Fig. 4:**
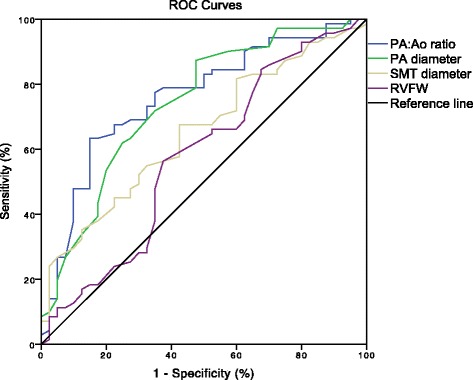
Receiver Operating Characteristics (ROC) curves of pulmonary artery to ascending aorta (PA:Ao) ratio, pulmonary artery (PA) diameter, septomarginal trabeculation (SMT) diameter and right ventricular free wall thickness (RVFW) with Areas Under the Curve of 0.759, 0.741, 0.652 and 0.576, respectively

By ROC analysis, a PA:Ao ratio of 0.83 was found to best detect mPAP ≥30 mmHg with a sensitivity of 73.2 % and a specificity of 67.5 %, and an area under the curve (AUC) of 0.759 (Fig. 4).

Measurements of PA:Ao ratio and PA diameter showed excellent inter-observer agreement with an Intra Class Correlation Coefficient (ICC) of 0.815 and 0.905 while ICCs for SMT diameters were only 0.563, and for RVFW 0.581.

### Cardiac outcomes

Patients were followed for 22.0 ± 14.9 months. During follow-up 40 cardiac events (34 hospitalizations for heart failure, 6 deaths from cardiovascular causes) occurred.

Table 4 shows the results of the multivariable Cox-regression model. By univariable analysis, SMT diameter (*p* = 0.023), RVFW (*p* = 0.034), PA diameter (*p* = 0.002), and PA:Ao ratio (*p* = 0.003) were significantly associated with cardiovascular events. After adjustment for age, sex, atrial fibrillation, diabetes, chronic obstructive pulmonary disease and coronary artery disease, PA diameter (*p* = 0.003), and PA:Ao ratio (*p* = 0.002) remained significant predictors of outcome. However, after multivariable adjustment, only PA:Ao ratio remained independently associated with event-free survival.

**Table 4:** Univariable, univariable adjusted, and multivariable adjusted Cox-regression model for cardiovascular events

	Regr.Coeff.	*p*-value	HR	95.0 % CI	
Univariable					
SMT area	0.663	0.069	1.941	0.950	3.963
SMT diameter	0.253	**0.023**	1.287	1.035	1.602
RVFW	0.282	**0.034**	1.326	1.022	1.720
PA diameter	0.093	**0.002**	1.097	1.035	1.163
PA:Ao x 100	0.027	**0.003**	1.027	1.010	1.046
RVEDV	0.003	0.365	1.003	0.997	1.009
RVEF	−0.016	0.340	0.985	0.954	1.017
Adjusted*					
SMT area	0.500	0.180	1.650	0.793	3.430
SMT diameter	0.233	0.060	1.262	0.990	1.608
RVFW	0.357	0.052	1.428	0.997	2.047
PA diameter	0.126	**0.003**	1.135	1.044	1.233
PA:Ao x 100	0.034	**0.002**	1.035	1.013	1.057
RVEDV	0.004	0.298	1.004	0.997	1.011
RVEF	−0.032	0.095	0.968	0.933	1.006
Multivariable, adjusted*					
PA:Ao x 100	0.031	**0.003**	1.031	1.010	1.053

*SMT* indicates septomarginal trabeculation, *RVFW* right ventricular free wall, *PA* pulmonary artery, *Ao* ascending aorta, *RVEDV* right ventricular end-diastolic volume

*Adjusted for age, sex, atrial fibrillation, diabetes, chronic obstructive pulmonary disease and coronary artery disease

**Fig. 5:**
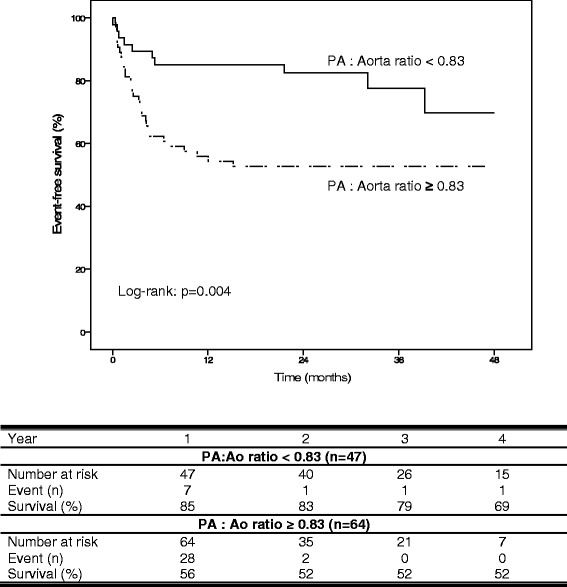
Kaplan-Meier analysis according to pulmonary artery to ascending aorta ratio. Patients with a pulmonary artery to ascending aorta (PA:Ao) ratio <0.83 showed a significantly better event-free survival than patients with a PA:Ao ratio ≥0.83

By Kaplan Meier analysis (Fig. 5), event-free survival was significantly worse in patients with a PA:Ao ratio ≥0.83 (log rank, *p* = 0.004).

## Discussion

The present study demonstrates that the PA:Ao ratio can be used as a simple and straight-forward indicator of PH associated with HFpEF. Moreover, the PA:Ao ratio is of prognostic value with respect to cardiac events.

In patients suffering from HFpEF right heart variables are of particular interest as they play an important role in terms of prognosis [5, 22–27]. Echocardiography is the first, and often only imaging modality used in HFpEF patients due to its wide availability. However, the RV is not easy to examine by echo and considerable inter- and intraobserver variability exists [9]. Furthermore, estimation of pulmonary hemodynamics may be inaccurate [8]. The definite diagnosis of PH demands invasive RHC to directly measure PA and wedge pressure, as well as trans-pulmonary and diastolic pulmonary pressure gradients. Although considered reasonably safe in experienced centers, RHC still carries a risk of morbidity and mortality [28]. Given the close link between pressures in the pulmonary circulation and RV dimensions and performance, several right heart imaging parameters have been shown to indicate the presence of PH [10, 13, 16, 26, 29–35]. CMR has been established as the gold-standard technique for the assessment of the right heart [10, 36]. It has the advantage that in addition to accurate and reproducible evaluation of heart size, function, and myocardial tissue characterization, CMR allows the assessment of the dimensions of the great arteries.

### Indices of right ventricular hypertrophy for the prediction of pulmonary hypertension

RV mass and RV mass to LV mass ratio (VMI) have been shown to correlate reasonably well with mPAP in previous studies [33, 34], but the estimation of RV mass from CMR images is time-consuming and limited by significant inter- and intra-observer variability [16, 32]. Furthermore, it requires dedicated software for off-line analysis.

RVFW thickness is much more easy to assess than RV mass or VMI. In a historical series this parameter appeared useful as surrogate marker of PH [30] and was also shown to be of prognostic value [31]. However, due to the hypertrabeculated nature of the RV considerable rater-dependent variation exists for this measurement, which significantly limits its usefulness [30]. In the present series, RVFW measurements showed weak reproducibility and did not differentiate between patients with and without moderate/severe PH.

The SMT is a prominent muscle band that originates from the basal ventricular septum and runs apical-laterally towards the RV free wall [37]. A recent retrospective CMR study on a small number of patients (*n* = 49) found that SMT mass could be a useful marker for the identification of PH [16]. However, in the present study SMT was hard to delineate in many patients, leading to significant rater-dependent variation. Its diagnostic power for the identification of patients with moderate/severe PH was clearly inferior as compared to PA:Ao ratio and PA diameter.

### Dimensions of the great arteries for the prediction of pulmonary hypertension

In contrast to the aforementioned parameters (RV myocardial mass, RVFW, SMT), the size of PA and Ao are easy to measure. Such measurements do not require dedicated software or advanced expertise. In the present study PA:Ao ratio was identified as the most potent predictor for the presence of moderate/severe PH. It correlated better with mPAP than PA diameter or indices of RV hypertrophy and showed excellent reproducibility. In addition, PA:Ao ratio was independently associated with event-free survival. From a clinical perspective the differentiation between isolated post-capillary PH and combined forms is crucial, in particular with regard to prognosis [21] and therapeutic consequences. The TPG and DPG have been introduced to quantify pulmonary vascular disease occurring as a consequence of PAWP elevation. In the present study, a greater PA:Ao ratio was predictive for TPG >12 mmHg.

Several previous studies have evaluated the usefulness of the PA:Ao ratio for the detection of PH [11–15]. However, only Chan and coworkers report data from a population with PH due to left heart disease [11]. In that retrospective analysis of 101 patients the authors defined a PA:Ao ratio cut-off of 0.84 for the detection of PH (mPAP >25 mmHg), which is in line with our results.

Other studies have used a PA:Ao ratio cut-off of >1.0 for the suspicion of PH. These included 60 patients with chronic obstructive pulmonary disease [13], 81 patients with connective tissue disease [15], 50 patients with mainly pulmonary disease [14], and a mixed population of 175 patients [12]. The differences with respect to the PA:Ao cut-off for the diagnosis of PH may be due to 1) retrospective design of all aforementioned studies, 2) heterogeneity of cut-offs for the diagnosis of PH (mPAP >20 mmHg [14] versus mPAP >25 mmHg [11–13, 15]), 3) heterogeneity of diagnoses underlying PH, and 4) small patient numbers [13–15]. Taken together, it remains to be determined whether the results of the present study are applicable in patient populations other than HFpEF.

### Limitations

Presented data have been collected in a single center setting. Therefore, a center-specific bias cannot be excluded. However, the major advantages of limiting data collection to a single center are 1. inclusion of a homogenous patient population, 2. adherence to a constant clinical routine, 3. constant quality of echocardiographic and CMR work-up and 4. constant follow-up.

PA and Ao diameters were acquired from axial HASTE black blood images and were not planned exactly in line with the vessel orientation. This may have led to some over- or underestimation of the vessel size.

The majority of patients included in this study were suffering from PH (83.8 % had mPAP ≥25 mmHg). Sixty four percent had at least moderate PH (mPAP ≥30 mmHg). Due to the small number of patients in the group without PH, patients were dichotomized in groups with mPAPs above and below 30 mmHg, defined as groups with no/mild PH versus moderate/severe PH. However, previous work shows that a PA:Ao ratio of 0.84 also discriminates between patients with mPAPs above and below 25 mmHg [11]. In addition to morphological indicators of right heart pressure overload, recent data indicate the feasibility of CMR to estimate pulmonary hemodynamics, such as mPAP and pulmonary vascular resistance by using numerical models [35]. Such estimates, however, include various assumptions. Furthermore, they require extensive experience of the investigator. Thus, the accuracy and widespread applicability of these measurements still need to be proven. Furthermore, we did not analyse RV mass, which might have augmented the diagnostic performance of the PA:Ao ratio. However, in the present work we aimed to identify parameters that are easy to measure and do not require dedicated software or extensive training.

## Conclusions

PA:Ao ratio is an easily assessable, straight-forward marker of PH in HFpEF patients. Moreover, it is strongly associated with outcome. Other CMR derived indices of RV pressure overload such as RVFW or SMT thickness are less accurate and reproducible.
